# Dietary Corn Silk (*Stigma maydis*) Extract Supplementation Modulate Production Performance, Immune Response and Redox Balance in Corticosterone-Induced Oxidative Stress Broilers

**DOI:** 10.3390/ani13030441

**Published:** 2023-01-28

**Authors:** Farid S. Nassar, Abdulaziz M. Alsahlawi, Hasan A. E. Abdellatif, Nancy N. Kamel, Ahmed O. Abbas

**Affiliations:** 1 Department of Animal and Fish Production, College of Agricultural and Food Sciences, King Faisal University, P.O. Box 420, Al-Ahsa 31982, Saudi Arabia; 2 Department of Animal Production, Faculty of Agriculture, Cairo University, P.O. Box 12613, Giza 12613, Egypt; 3 Department of Finance, College of Business Administration, King Faisal University, P.O. Box 420, Al-Ahsa 31982, Saudi Arabia; 4 Department of Internal Medicine, Faculty of Medicine, Al-Azhar University, P.O. Box 71511, Assuit 71524, Egypt; 5 Department of Animal Production, National Research Center, El Buhouth St., Dokki, P.O. Box 12622, Giza 12622, Egypt

**Keywords:** broiler, production performance, immune response, stress markers, redox status, corn silk extract

## Abstract

**Simple Summary:**

Modern poultry production is constantly confronting stressful conditions which jeopardize profitability. Under stress conditions, the increasing level of stress hormone corticosterone induces immunosuppression and negatively influences the redox status, which increases morbidity, disturbs physiological hemostasis, and reduces production performance. Corn silk is an agro-byproduct that is rich in bioactive antioxidants and anti-inflammatory compounds. Thus, the present study investigated the potential use of corn silk extract to alleviate the negative impact of corticosterone-induced oxidative stress and immunosuppression. The results demonstrated the negative impacts of corticosterone administration on broilers performance, immune response, and redox status. However, corns silk extract supplementation with its high total phenolic-content modulated the immune response and redox balance in corticosterone-induced-stress birds. It can be concluded that, under stress rearing conditions, dietary corn silk extract supplementation can alleviate the negative impacts of stress induced by corticosterone exposure.

**Abstract:**

Immunosuppression is a serious consequence of oxidative stress exposure that negatively affects the productivity and profitability of birds, as well as their well-being. Thus, the present investigation was designed to evaluate the potential of corn silk extract (CSE) supplementation to overcome the negative impacts of oxidative stress induced by corticosterone administration (CORT) in broiler chickens. A total of 280 one day old Cobb 500 male chicks were divided into four groups in 2 × 2 factorial arrangements. The experimental groups included CSE supplementation (0 or 500 mg/kg diet, from 20 to 35 days of age) and CORT administration (0 or 25 mg/kg diet, from 22 to 35 days of age) as independent factors. At the end of week five of age, production performance parameters were measured. The humoral and cell-mediated immune response parameters, redox status, and stress markers were determined. Data revealed deleterious effects of CORT administration on the broilers’ body weight, body weight gain, and feed conversion ratio. Moreover, an exponential increase in stress marker levels, in addition to immunosuppression and redox imbalance, were associated with CORT administration. However, CSE supplementation, with its high total phenols content, partially alleviated the negative impacts of CORT administration, as shown by a significant improvement in immune response parameters and antioxidant activity, as well as a reduction in stress marker levels. Furthermore, CSE supplementation to non-stressed birds even significantly improved total antioxidant activity, total white blood cells (TWBCs) count, T-lymphocyte stimulating index, and wattle thickness. It can be concluded that, under stress conditions in commercial broiler farms, dietary CSE supplementation can strongly be recommended to modulate the negative impacts of stress. Therefore, CSE can be used as an effective immunomodulator and antioxidant agent to increase commercial broiler farm productivity and profitability.

## 1. Introduction

During the course of normal respiration process and oxygen metabolism in living cells, reactive oxygen species (ROS) are constantly formed and reduced to water. The low production level of ROS is important to stimulate endogenous antioxidant defense mechanisms that protect cells from oxidative damage [1]. However, oxidative stress arises when ROS production surpasses the removing capacity of the endogenous antioxidant system. As a consequence, ROS accumulation leads to several biological damage and pathological conditions that hinder poultry growth and impair productivity [2,3]. Currently, the modern poultry production system is subjected to several technological, environmental, nutritional, and internal stress factors, which compromise productivity and subsequently profitability [4,5]. 

Stress response, a mechanism by which the body restores homeostasis under any given stressor, is initiated by a stress exposure which triggers and activates the hypothalamic-pituitary-adrenal (HPA) axis. Upon HPA axis activation, a substantial increase in corticosterone hormone (CORT) circulation levels occur, which mediates a number of physiological beneficial changes, in order to sustain body functions and help in restoring homeostasis [6,7]. However, chronic stress exposure, owing to long-term CORT release, reverses the beneficial effects of CORT leading to a broad range of metabolic disorders and disease states [8]. Metabolic changes, such as the inhibition of skeleton muscles protein synthesis and lipid metabolism disorder on one hand, and immunosuppression on the other hand, are common observations in physiological stress responses induced by endogenous high CORT release or exogenous CORT administration [9,10]. Post, et al. [11] stated that the addition of the CORT hormone to broiler chickens may serve as a practical substitution to investigate hormone-related stress to circumvent animals treated by physical or physiological stressors. Moreover, physiological stress mediated through the administration of CORT was reported to induce impairment in the immune defense of small intestinal mucosa in broiler chickens challenged with *Clostridium perfringens* [12]. In a comprehensive study on the effect of CORT injection on broiler chickens’ productivity, immunity, and cell death, Mehaisen, et al. [13] demonstrated that CORT administration negatively affects broiler performance, induces immunosuppression, and initiates cell apoptosis. Stress exposure directly impairs immune responses through reducing the growth of the primary lymphoid organs, which consequently reduce the number and activity of T- and B-lymphocytes [6]. However, to overcome the negative impacts of oxidative stress at the cellular level, as well as restoring redox balance and optimizing immune responses, an exogenous antioxidant supplementation seems to be a promising strategy. 

Several nutritional strategies have recently been proposed to subdue the negative impacts of oxidative stress and its subsequent inflammation response, in order to improve poultry productivity and well-being [14]. One of the most promising, safe, straightforward, and cost-effective strategies is the use of nutraceuticals, which include medicinal herbs and herbal extracts, in order to maintain optimum poultry production under different stress conditions [15]. Recently, phytochemicals, a non-nutritive plant-derived compound, were intensively presented as potential, potent anti-oxidant, and anti-inflammatory agents that can safely be supplemented to stressed-birds diet [1,2,16,17]. *Stigma maydis,* or corn silk, is a stigma of the maize female flower that is rich in phenolic compounds, particularly flavonoids, alkaloids, and saponins, and has been utilized as a nutraceutical for a wide range of diseases [18,19]. Antioxidant activity and anti-inflammation effects were among the reported bioactive properties of corn silk extracts (CSE) [18,20,21]. Vranjes, et al. [21] concluded that corn silk can be used as a natural antioxidant source for the prevention and treatment of different oxidative stress-induced diseases. However, the previous work mainly focused on the in vitro CSE properties, whereas the in vivo biological potential is not fully studied. Therefore, the present study was designed to investigate the potential of CSE supplementation in mitigating the negative impacts of the CORT-induced stress model on broiler immune response and redox status, as well as production performance. In addition, an economic study was conducted to apprehend the losses originated from oxidative stress induction and the potential beneficial effect of CSE supplementation to retrieve profitability. 

## 2. Materials and Methods

### 2.1. Corn Silk Total Phenolscontent and Phenolic Profile

The corn silk extract was prepared according to the method described by Abbas, et al. [22]. The corn silk total phenols were determined using colorimetric method by the utilization of the Foli-Ciocalteu reagent according to the method described by Singleton and Rossi [23]. The total phenolic content was calculated following the regression equation of the standard plot: (y = 998.88x + 0.9291; r^2^ = 0.9998). The result was expressed as mg gallic acid (Product No, 27645; Sigma-Aldrich, St. Louis, MO, USA) equivalent per 100 g dry sample (mg GAE/100 g). The absorbance of sample was quantified using a spectrophotometer (UV/Vis Spectrophotometer, Jenway, England). The corn silk total phenols content is presented in Table 1.

Fresh corn silk sample was collected, air dried and grinded to fine powder. A 2.0 g of corn silk powder was accurately weighed and put into a clean 15 mL glass tube. First, 5 mL of ethanol/water 70/30 (V/V) was added and the tube was vortexed for exactly 1 min. Next, the tube was put in ultrasonic bath for 15 min at room temperature for further extraction. The sample was then centrifuged at 5000 rpm for 25 min. Finally, the supernatant was filtered through a 1-mL plastic syringe with Captiva Premium Syringe Filters (p/n 5190–5107) and then injected into the HPLC system. 

The phenolic compounds profile was screened and quantified using HPLC equipment (Agilent1260 infinity HPLC Series, Agilent, Santa Clara, CA, USA), equipped with Quaternary pump. The used column was aKinetex^®^1.7µm EVO C18 50 mm × 2.1 mm, (Phenomenex, Torrance, CA, USA), operated at 30 °C. The separation was achieved using a binary elution gradient with HPLC grade water 0.1% H_3_PO_4_ (*v*/*v*) and acetonitrile 0.1% H_3_PO_4_ (*v*/*v*). The flow rate was set at 0.2 mL/min and the injected volume of the extract was 20μL. Detection was performed using variable wavelength detector (VWD) set at 280 nm. The phenolic compounds were quantified against HPLC grade standards (Sigma-Aldrich, St. Louis, MO, USA), and the result is presented in Table 1.

### 2.2. Experimental Design and Birds’ General Management

A total number of 280 one day old male Cobb 500 chicks were recruited and reared in closed-system floor house chamber under similar environmental conditions. The experimental groups were assigned to four symmetric experimental groups (seven replicates × 10 birds) in 2 × 2 factorial arrangements, with CSE-supplemented groups (0 or 500 mg/kg diet, from 20 to 35 days of age) and CORT-administrated groups (0 or 25 mg/kg diet, Cat#: C2505; Sigma Aldrich, St. Luis, MO, USA, from 22 to 35 days of age) served as the primary factors. Two groups were fed the basal diet and the other two groups were fed the basal diet supplemented with 500 mg CSE per kg. The four experimental groups were as follow: (1) non-stressed group with no CSE supplementation (NS − CSE), (2) non-stressed group with CSE supplementation (NS + CSE), (3) CORT-induced stress group with no CSE supplementation (CORT − CSE), and (4) CORT-induced stress group with CSE supplementation (CORT + CSE). The basal diet was formulated to cover the nutritional requirements following the NRC [24] and the management guide of Cobb 500 broiler and was offered in a mash form (Table 2). Birds had free access to feed and water at all times. Each replicate was reared in a one m^2^ floor pen with five cm deep litter of hardwood shavings.

### 2.3. Production Performance

Body weight (BW) was recorded at the beginning of the third week and at the end of the fifth week of age for each group replicate. Feed intake was recorded per each group replicate starting from week three to week five of age. The daily weight gain (DWG) and feed conversion ratio (FCR) were then calculated.

### 2.4. Blood Sampling and Preparation

At the end of the fifth week of age, 5 mL blood samples were collected from seven birds from each group (one bird per replicate) in heparinised tubes. One mL of each sample was used to count total white blood cells (TWBCs) and to calculate heterophils to lymphocytes (H/L) ratio. Meanwhile, the rest of the blood sample was centrifuged at 2000× *g* and 4 °C for 10 min, then plasma were collected and stored at −20 °C for further analysis.

At the same time, another seven blood samples from each group were collected in a heparinised tubes and used to isolate the peripheral blood mononuclear cells (PBMCs) as Mehaisen, et al. [13] described for malondialdehyde (MDA), tumor necrosis factor-α (TNF-α) and heat shock protein 70 (HSP-70) quantification and T- and B-lymphocytes proliferation assessment. Briefly, the PBMCs were firstly washed twice using RPMI 1640 medium (Thermo Fisher Scientific, Waltham, MA, USA), and re-suspended with phosphate buffer saline (PBS) (pH 7.2). Next, a 1 mL of cell suspension was centrifuged at 1030× *g* for 20 min. Then, the aggregated granules were obtained and stored at −70 °C for further analyses.

### 2.5. Redox Status and Stress Markers Evaluation

#### 2.5.1. Plasma Total Antioxidant and Antioxidant Enzymes

The total antioxidant activity (TAC) and superoxide dismutase (SOD) enzyme activity were determined in blood plasma using colorimetric assay kits following the manufacturer protocol (Cat #: MBS2540515 and MBS9718960, respectively; MyBioSource, San Diego, CA, USA). Meanwhile, glutathione S-transferase (GST) activity and glutathione (GSH) concentration were measured using colorimetric kits (Cat#: A004 and A006, respectively; Nanjing Jiancheng Bioengineering Institute, Nanjing, China).

#### 2.5.2. Stress Biomarkers

The previously collected and stored PBMCs were washed and re-suspended in 1 mL of PBS. The suspension was kept on ice for one min and sonicated for another one min. The homogenate was then centrifuged at 1030× *g* and 4 °C for 15 min to obtain the supernatant, where MDA, TNF-α and HSP-70 concentrations were quantified. The malondialdehyde (MDA) was estimated as an indicator of lipid peroxidation in PBMC using a colorimetric assay kit (Cat #: MBS9718963; MyBioSource, San Diego, CA, USA). Meanwhile, the PBMCs levels of TNF-α and HSP-70 were quantified using chicken ELISA kits (Cat #: MBS2509660 and MBS2702636, respectively; MyBioSource, San Diego, CA, USA). According to the manufacturer, the intra-assay and inter-assay coefficients of variations percentages (CV%) were 5.57 and 5.89% for the TNFα, and <10% and <12% for HSP70, respectively. The detection ranges were 31.25–2000 pg/mL for TNF-α, and 0.312–20 ng/mL for HSP-70. Further, plasma corticosterone concentration was measured using a commercial chicken specific ELISA kit (Cat #: MBS701668; MyBioSource, San Diego, CA, USA). According to the manufacturer, the intra- assay and inter-assay CV% were ˂8 and ˂10%, respectively. The assay sensitivity was >0.5 ng/mL, with a dynamic detection range of 0.5 to 20 ng/mL.

### 2.6. Immune Response Parameters

#### 2.6.1. TWBCs Count and H/L Ratio Determination

Total white blood cells (TWBCs) count was performed in whole blood samples for each group (*n* = 7; one sample per replica) according to the methods described by Gehad, et al. [25]. Whole blood sample (10 µL) was mixed with brilliant cresyl blue dye (490 µL), and then the total leukocytes were counted using a hemocytometer slid and a light microscope at a magnification of 200×. Meanwhile, leukocytes differentiation and calculation were performed according to Abass, et al. [26].

#### 2.6.2. Sheep Red Blood Cells Antibody Titer

The antibody titer against sheep red blood cells (SRBCs) was measured as a humoral-mediated immune response parameter. At day 35 of age, seven birds from each experimental group (one bird per replica) were randomly chosen and intravenously injected with 1 mL of 5% SRBCs saline suspension. A week later, blood samples were collected from each bird and serum was separated by centrifugation at room temperature and 220 ×g for 10 min. The SRBCs antibody titer was then quantified in serum using a micro-hemagglutination assay [27]. The SRBCs antibody values were expressed as log2 of the reciprocal of the highest dilution giving complete agglutination.

#### 2.6.3. Peripheral T- and B-Lymphocytes Proliferation

T- and B-lymphocytes proliferations were measured as the stimulating index (SI) according to Mehaisen, et al. [28]. Firstly, the collected PBMCs were washed using RPMI 1640 culture medium (Thermo Fisher Scientific, Waltham, MA, USA). Next, the viable lymphocytes cells were detected using Trypan Blue dye (Sigma-Aldrich, MO, USA). Then, the viable cells were plated, in a 96-well plate, at a concentration of 6 × 10^6^ cells per well. Afterwards, 50 μL of concanavalin-A at a concentration of 45 μg/mL (Sigma-Aldrich, St. Luis, MO, USA) was added to stimulate T-cells proliferation. Meanwhile, 50 μL of lipopolysaccharide at a concentration of 10 μg/mL (Sigma-Aldrich, St. Luis, MO, USA) was added to stimulate B-cells proliferation. The un-stimulated control cells were supplemented with 50 μL of RPMI-1640 medium. After that, cells were incubated for two days at 42 °C and 5% CO_2_. Subsequently, 15 μL of tetrazolium salt (MTT) (Sigma-Aldrich, St. Luis, MO, USA) was added to each well at a concentration of 5 mg/mL, and then cells were incubated for another four hours at 42 °C. Later after incubation, 100 μL of 10% sodium dodecyl sulfate dissolved in 0.04 M HCl, was added. Finally, the absorbance was detected using a microplate reader at 570 nm, and the T- and B-lymphocytes stimulating indexes were calculated as the ratio between optical densities of stimulated cells to the un-stimulated control cells.

#### 2.6.4. Wattle Swelling Test

Broiler wattle swelling induced by phytohemagglutinin (PHA-P) mitogen intradermal injection was evaluated as cell-mediated immune response assessment test following the protocol described by Abass, et al. [26]. Briefly, seven birds from each experimental group (one chicken per replica) were injected with PHA-P (Sigma-Aldrich, St. Luis, MO, USA) dissolved in sterile PBS buffer (100 µg: 0.1 mL; *w*/*v*) in the left wattle. The wattle thickness diameter in response to the PHA-P injection was calculated as the difference between the thickness existing immediately before and 24 h after PHA-P injection in mm.

### 2.7. Economic Efficiency Calculations

The economic efficiency was evaluated according to the current market prices in Saudi Arabian riyal (1 US dollar = 3.76 SAR). The feed, water, chicks, labor, vaccination, veterinarian care and transportation were set as the economic inputs calculated per bird according to the market costs and prices. The total revenue was calculated per bird as the sum of chicken price and manure revenue. The profit was calculated as the difference between total revenue and total costs per bird. The economic efficiency was calculated as the profit divided by the total costs. Finally, the relative economic efficiency was calculated for each experimental group relative to the NS − CSE group by dividing the economic efficiency of the experimental group on the economic efficiency of the NS − CSE group multiplied by 100.

### 2.8. Statistical Analysis

The production performance, immune response, redox and stress markers data were normally distributed with homogeneity of variances and were subjected to two-way analysis of variance (ANOVA) using the general linear model of SAS (SAS^®^ 9.1.3; SAS Institute Inc., Cary, NC, USA). The main effects of CSE supplementation, CORT admission and their interaction (CSE×CORT) were assessed. Duncan Multiple Range post hoc test was performed to determine significance among the experimental group means. Results were expressed as mean ± SEM and the post hoc test was conducted at 95% confidence level (*p* < 0.05).

## 3. Results

### 3.1. Production Performance

The production performance parameters of broiler chickens fed corn silk extract (CSE) and subjected to cotricosterone-induced-oxidative stress (CORT) are presented in Table 3. A significant deterioration in final body weight (BW), daily weight gain (DWG), and feed conversion ratio (FCR), were observed in CORT-induced stress groups. At the fifth week of age, the BW and DWG of broilers in the CORT-CSE group were decreased by 33 and 52%, respectively, compared with the NS−CSE group. Meanwhile, CSE supplementation to the CORT + CSE group was able to significantly restore 18 and 28% of the decreased final BW and DWG, respectively, compared with the NS − CSE group. The feed conversion ratio (FCR) was significantly impaired in the CORT − CSE group compared with the NS−CSE group; 2.98 vs. 1.60, respectively. However, FCR was significantly improved in the CORT + CSE compared to the CORT − CSE group, 2.10 vs. 2.98, respectively. A significant interaction between CSE supplementation and CORT administration for BW, BWG, and FCR, was noticed. Meanwhile, under non-stress-rearing condition, no significant effects were noticed between the CSE supplemented group and the NS−CSE group regarding different production performance parameters.

### 3.2. Stress Markers and Redox Status

The stress markers and redox parameters of broiler chickens fed CSE and subjected to CORT induced-oxidative stress are presented in Table 4. The CORT administration to the CORT − CSE group exponentially increased the MDA, TNF-α, and HSP-70 levels, as well as plasma corticosterone concentration by 4.6, 2.0, 3.4, and 1.9-fold, respectively, compared with the NS − CSE group. On the contrary, the antioxidant enzymes (i.e., SOD and GST) and total antioxidant capacity (TAC) activities, as well as GSH concentration, were significantly reduced in the CORT − CSE group compared with the NS−CSE group. All these physiological changes in stress markers levels and the antioxidant activities demonstrate the oxidative stress induction by CORT administration. Meanwhile, CSE supplementation to the CORT + CSE group alleviated the negative oxidative stress impacts of CORT administration with a significant reduction in the MDA, TNF-α, and HSP-70 levels, by 1.8, 1.2, and 1.6-fold, respectively, while significantly elevating the SOD, GST, and TAC activities by 22, 30, and 24%, respectively, compared with the CORT − CSE group. Furthermore, the CSE supplementation to the NS + CSE group broiler chickens significantly improved the SOD and TAC activities and increased GSH concentration compared with the NS − CSE group. A significant interaction between CSE supplementation and CORT administration was detected for MDA, HSP-70, and corticosterone concentrations, as well as TAC activity.

### 3.3. Innate and Acquired Immune Responses

The immune responses of broiler chickens fed CSE and subjected to CORT induced-oxidative stress are presented in Table 5. Data revealed serious deterioration in all measured immune response parameters. The TWBCs count was decreased by 1.8-fold, while H/L ratio increased by 2.3-fold in the CORT − CSE group compared with the NS − CSE group. Furthermore, T- and B-lymphocytes stimulating indexes significantly decreased by 2 and 3-fold, respectively, in the CORT − CSE group compared with the NS − CSE group. In addition, SRBC antibody formation showed 50% decrease in the CORT − CSE group compared with the NS − CSE group. Wattle swelling in response to PHA-antigen injection was significantly suppressed in the CORT − CSE group compared with the NS − CSE group. However, CSE supplementation to the CORT + CSE group was able to partially restore innate immune response as well as cell-mediated and humoral immune function. Furthermore, CSE supplementation to the NS + CSE group showed significant effect on TWBCs count T-lymphocyte proliferation and PHA mitogen response. A significant interaction between CSE supplementation and CORT administration was observed concerning H/L ratio, B-lymphocytes stimulating index and wattle thickness.

### 3.4. Economic Efficiency

The economic efficiency of CSE supplementation to CORT induced-oxidative stress broilers are presented in Table 6. The present economic evaluation demonstrated a serious drop in the economic efficiency in the CORT − CSE group relative to the NS − CSE group. Meanwhile, CSE supplementation to the CORT + CSE group partially improved economic efficiency, which consequently shrank the revenue loss. Furthermore, CSE supplementation to broilers reared under non-stressed conditions increased the relative economic efficiency by 18% compared with the non-stressed CSE.

## 4. Discussion

Physiological stress induced by CORT injection was presented as a valid stress model for studying various stress impacts and possible interventions in broiler chickens [12,17,29,30]. The present results indicated a significant decrease in production performance with low final BW, DWG, and FCR impairment for CORT-administered birds. Short term pre-slaughter CORT injection reported to increase live BW loss with breast muscle oxidative injury generation in broilers [17]. Abeddargahi, et al. [31] reported a significant reduction in dry matter digestibility and impairment in FCR for broiler chickens injected with CORT. The administration of CORT for 28 days starting from day 15 to 42 of age, at a level of 30 mg/kg diet, significantly reduced broiler chickens BW, body weight gain, and FI, while increasing FCR and mortality rate [29]. Jiang, et al. [32] linked between the reduction in BWG and FI with the increase in FCR to the increasing apoptotic index in the broiler intestinal epithelial cells. The observed improvement in production performance with CSE supplementation to CORT-induce oxidative stress may be mediated through reducing oxidative stress and inflammation, as well as changing in the gut microbiome community promoted by the gut-brain axis [33].

The main avian immune system components are innate and adaptive immunity [34,35,36]. Besides, adaptive immunity is divided into cell-mediated and antibody-mediated (humoral) responses, which are responsible for intracellular and extracellular antigen elimination, respectively [37]. Corticosterone administration negatively influences all aspects of broiler immune responses. Avian Leukocytes, or TWBCs, are a group of cells located in the blood that are considered as a part of the body’s immune system [38]. The decrease shift observed in TWBCs count in response to CORT administration reflects the suppression of the innate immunity. Furthermore, under stress conditions, the number of circulating heterophils substantially increases; thus, a shift in leukocytes population occurs, which is marked by the increase in H/L ratio that has been used as a sensitive stress marker [7,39,40,41]. The observed shift in leucocyte cells population and type can be explained as a result of CORT administration, which consequently increased the blood circulation level of corticosterone [7]. Corticosterone injection to broiler chickens reported to induce a reduction in TWBCs count with an increase in H/L ratio [13]. An increased shift in H/L ratio was reported in CORT injected broilers [31]. Stress induced by ACTH administration for seven days significantly reduced lymphoid organ weight, (i.e., thymus, bursa, and spleen) in 6 weeks old chickens [39]. Further, stress induced by seven days CORT injection caused a reduction in both primarily (thymus and bursa) and secondary (spleen) lymphoid organs relative weights in broiler chickens [13]. Corticosterone injection also reported to reduce the relative weights of bursa and the lymphocyte density in the bursa medulla in broiler chickens [31]. Post, et al. [11] reported a significant inhibition of SRBC specific antibody production in broiler chickens treated with CORT at 10 or 20 mg/L of drinking water. The observed reduction in blood TWBCs and lymphocytes proliferation indexes as well as the reduction of antibody production against SRBC associated with CORT administration can partially be attributed to the decrease in lymphoid organs weight and their impaired functions in response to stress exposure [6]. Further, a depression of antibody titer against SRBC, low plasma immunoglobulin concentrations, and a reduction of lymphocyte proliferation were found to be associated with an increase in the plasma corticosterone levels and an inhibition of leukocyte protein synthesis in broilers subjected to chronic heat stress [42].

In order to determine the onset of oxidative stress, biomarkers in blood have been used. Ducatelle, et al. [43] defined biomarkers as “measurable alteration in biological substances that is associated with normal or abnormal conditions”. In case of oxidative stress, the increase in the blood MDA and the decrease in the antioxidant enzymes activity were used as markers for evaluating oxidative stress in vivo [3,44,45]. In the present study, the drop in the antioxidant activity and the increase in MDA formation were detected, which reflect the onset of oxidative stress and the imbalance of birds’ redox status. Hu and Deng [46] reported that corn silk flavonoids extract inhibited lipid peroxidation (MDA) and increased antioxidant enzymes SOD and glutathione peroxidase (GSH-Px) levels in exercise-induced oxidative stress mice. Furthermore, the increasing levels of the pro-inflammatory cytokines, such as TNF-α, secreted during the normal course of stress induce severe oxidative stress as well as participate in the stimulation of corticosterone secretion through activating the HPA axis, which finally leads to immunosuppression [16]. A significant association between an elevation of blood corticosterone concentration and the HSP-70 over-expression was reported in broilers subjected to feed restriction stress [47], heat stress [48], and CORT-induced stress [49]. The plasma corticosterone concentration significantly increased with CORT administration, which consequently led to the observed immunosuppression response. Corn silk flavonoid extraction reported to possess many potential biological characteristics, such as an antioxidant effect [50]. Furthermore, Zhang, et al. [51] stated that corn silk polysaccharides extract is characterized by in vitro antioxidant activities that enhanced glutathione peroxidase, SOD, and TAC activities, while lowering MDA levels in both the serum and liver of Kunming mice in vivo. Such antioxidant activity potential of CSE might be the key modulator of the negative effects of CORT-induce oxidative stress that played a vital role as an immunomodulator and antioxidant booster.

Several research studies demonstrated that corn silk is a rich source of total phenols and total flavonoids [18,19,21,46]. The performed phenolic profile analysis following HPLC technique revealed that CSE is rich in a number of phenolic compounds. The most abundant phenolic compounds found were myricetin, chlorogenic acid, rutin, ferulic, and syringic acid. These phenolic compounds were reported to exert strong antioxidant activity. Myricetin reported to have antioxidant and anti-inflammation properties in addition to a protection against lipid peroxidation as well as preventing NF-κB activation in a monocyte model [52]. Moreover, myricetin was found to decrease TNF-α and MDA while significantly increase the activities of SOD and GSH-Px [52]. Furthermore, rutin, one of the active constituents found in CSE, was found to alleviate the oxidative stress induced by lipopolysaccharide through inhibition of MAPK–NF-κB activation and increase antioxidant SOD, catalase, glutathione peroxidase, and heme oxygenase-1 enzymes activity [53]. Other bioactive properties of rutin were reviewed, including antioxidant, cytoprotective, anticarcinogenic, neuroprotective, and cardioprotective activities, which approximately all suggested to be mediated by its antioxidant properties [54]. Zduńska, et al. [55] reviewed that ferulic acid has strong antioxidant properties, by acting as a free radical scavenger, a free-radical-catalyze-enzymes inhibitor, and an antioxidant-enzymes-activity enhancer. Additionally, syringic acid is reported to have an anti-inflammatory activity as well as strong free radical scavenging activity due to the presence of two methoxy moieties [56]. From the above mentioned information, CSE contains a number of phenolic compounds that have strong antioxidant and anti-inflammation activities. These bioactive compounds are directly responsible for the observed reduction of stress markers levels as well as the improvement in the redox status and immune response of CORT-induced oxidative stress broilers. Thus, CSE can safely be used to alleviate the oxidative stress and reduce inflammation in broiler chickens.

## 5. Conclusions

The current study investigated the anti-stress potentials of CSE against CORT-induced oxidative stress on broiler chickens’ immune response, production, and redox status. The deleterious effects of CORT administration on innate and acquired immune responses, as well as DWG and FCR, were substantial. Imbalance redox status was noticed with CORT administration, accompanied by elevated MDA level, and reduced TAC and antioxidant enzymes activities. However, CSE was found to contain a considerable amount of total phenols, which is known to have antioxidant and anti-inflammation properties. Myricetin, chlorogenic acid, rutin, ferulic, and syringic acid, are some of the CSE detected bioactive phenolic compounds. The supplementation of CSE to CORT-induced oxidative stress broiler chickens, owing to its antioxidant activity, was able to retrieve redox balance and to improve production performance. The CSE supplementation showed an immunomodulation effect as well as an antioxidant activity enhancement. Furthermore, CSE supplementation to non-stressed broilers significantly improved the cell-mediated immune response as well as increased the antioxidant activity. Thus, it can be inferred that dietary CSE supplementation can efficiently be used to alleviate the negative impacts of oxidative stress in broilers reared under commercial stress conditions. In addition, CSE supplementation can work as an immunomodulation and antioxidant booster to increase commercial broiler chickens’ profitability.

## Figures and Tables

**Table 1:** Phenolic compounds and total phenols of corn silk extract.

Phenolic Compound	Amount *
Myricetin	60.14
Chlorogenic acid	42.85
Rutin	33.35
Ferulic	31.71
Syringic acid	24.52
o-Cumaric	7.11
Quercetin	2.37
p-hydroxybenzoic	1.20
Cinnamic	1.14
Kaempferol	0.85
p-Coumaric	0.58
Total phenols **	460.50

* The amount of corn silk phenolic compounds was measured by HPLC and presented as mg per kg on dry matter basis. ** Corn silk total phenols was measured as mg Gallic acid equivalent/100 g sample.

**Table 2:** Ingredients and nutrient composition of the basal diets fed from days 20 to 35 of age.

Ingredients	g/kg
Corn	626
Gluten meal	20.0
Soybean meal, 48%	292
Soya oil	25.0
Di-calcium phosphate	16.5
Limestone	7.00
Salt	4.50
Premix *	5.00
L-threonine	0.50
DL-methionine	0.80
L-lysine	1.70
Choline chloride	0.20
3-Phytase	0.80
**Calculated analysis**	
Calcium	8.48
Phosphorus	4.21
DL-methionine	5.68
L-lysine	11.00
Sodium	1.40
**Chemical analysis**	
Metabolizable energy, kcal/kg	3150
Crude protein, %	20.2
Crude fat, %	5.88
Ash, %	0.56

* Premix provided the following vitamins and minerals per kg of diet: vit A: 1500 IU; vit D3: 200 IU; vit E: 10 mg; vit K3: 0.5 mg; thiamine: 1.8 mg; riboflavin: 3.6 mg; niacin: 35 mg; pantothenic acid: 10 mg; pyridoxine: 3.5 mg; biotin: 0.15 mg; folic acid: 0.55 mg; cobalamin: 0.01 mg; Fe: 80 mg; Mn: 60 mg; Zn: 40 mg; Cu: 8 mg; I: 0.35 mg; Se: 0.15 mg.

**Table 3:** Production performance parameters of broiler chickens subjected to corticosterone induced-oxidative stress (CORT) and fed corn silk extract (CSE).

Parameter	Non-Stress		CORT-Induced Stress		SEM	*p*-Value		
	−CSE	+CSE	−CSE	+CSE		CSE	CORT	CSE × CORT
Intial BW, g	731.4	741.4	738.6	729.3	18.36	0.9846	0.8928	0.6042
Final BW, g	2059 ^a^	2113 ^a^	1374 ^c^	1749 ^b^	42.94	<0.0001	<0.0001	0.0010
DWG, g	94.80 ^a^	97.96 ^a^	45.36 ^c^	72.81 ^b^	3.44	0.0002	<0.0001	0.0017
FI, g	2121	2179	1901	2141	117.2	0.2161	0.2823	0.4413
FCR	1.60 ^c^	1.58 ^c^	2.98 ^a^	2.10 ^b^	0.07	<0.0001	<0.0001	<0.0001

Means with different superscript letter differ significantly (*p* < 0.05). Initial BW: body weight at week 3 of age; final BW: body weight at week 5 of age; DWG: daily weight gain; TWG: total weight gain; FI: feed intake during the 4th and 5th weeks of age; FCR: feed conversion ratio.

**Table 4:** Stress markers and redox parameters of broiler chickens fed corn silk extract (CSE) and subjected to corticosterone-induced oxidative stress (CORT).

Parameter	Non-Stress		CORT-Induced Stress		SEM	*p*-Value		
	−CSE	+CSE	−CSE	+CSE		CSE	CORT	CSE × CORT
MDA, nmol/mL	1.02 ^c^	0.97 ^c^	4.69 ^a^	2.66 ^b^	0.18	<0.0001	<0.0001	<0.0001
TNF-α, pg/mL	93.7 ^c^	85.0 ^c^	185.6 ^a^	160.7 ^b^	4.90	0.0022	<0.0001	0.1125
HSP-70, ng/mL	25.43 ^c^	21.71 ^c^	84.71 ^a^	52.71 ^b^	2.95	<0.0001	<0.0001	<0.0001
CORT, ng/mL	4.43 ^c^	4.16 ^c^	8.63 ^a^	6.50 ^b^	0.25	<0.0001	<0.0001	0.0009
SOD, U/mL	4.92 ^b^	5.87 ^a^	2.92 ^d^	3.55 ^c^	0.19	0.0003	<0.0001	0.4022
GST, U/mL	4.73 ^a^	5.17 ^a^	3.11 ^c^	4.04 ^b^	0.17	0.0004	<0.0001	0.1508
GSH, μg/mL	0.73 ^b^	0.86 ^a^	0.56 ^c^	0.61 ^c^	0.02	0.0010	<0.0001	0.1058
TAC, U/mL	8.75 ^b^	12.01 ^a^	5.44 ^d^	6.72 ^c^	0.41	<0.0001	<0.0001	0.0227

Means with different superscript letter differ significantly (*p* < 0.05). MDA: malondaialdhyd; TNF-α: tumor necrosis factor alpha; HSP-70: heat shock protein 70; CORT: corticosterone; SOD: superoxide dismutase; GSH: Glutathione; GST: Glutathione S-transferase; TAC: total antioxidant capacity.

**Table 5:** Immune response related parameters of broiler chickens fed corn silk extract (CSE) and subjected to corticosterone-induced oxidative stress (CORT).

Items	Non-Stress		CORT-Induced Stress		SEM	*p*-Value		
	−CSE	+CSE	−CSE	+CSE		CSE	CORT	CSE × CORT
TWBCs, × 10^3^/mL	49.01 ^b^	55.52 ^a^	26.91 ^d^	35.64 ^c^	1.64	0.0001	<0.0001	0.5049
H/L ratio	0.40 ^c^	0.31 ^c^	0.92 ^a^	0.63 ^b^	0.04	<0.0001	<0.0001	0.0081
SI T-lymphocytes	4.98 ^b^	5.90 ^a^	2.52 ^c^	3.05 ^c^	0.23	0.0046	<0.0001	0.4204
SI B-Lymphocytes	2.81 ^a^	3.15 ^a^	0.87 ^c^	1.79 ^b^	0.12	<0.0001	<0.0001	0.0240
SRBC, log_2_	7.7 ^a^	8.1 ^a^	3.7 ^c^	5.1 ^b^	0.29	0.0043	<0.0001	0.1025
Wattle thickness, mm	0.34 ^b^	0.47 ^a^	0.25 ^d^	0.31 ^c^	0.01	<0.0001	<0.0001	0.0011

Means with different superscript letter differ significantly (*p* < 0.05). TWBCs: total white blood cells; H/L ratio: heterophils to lymphocytes ratio; SI: stimulating index.

**Table 6:** Economic efficiency of broiler chickens fed corn silk extract (CSE) and subjected to corticosterone-induced oxidative stress (CORT).

**Economic Values**	**Non-Stress**		**CORT-Induced Stress**	
	**−** **CSE**	**+CSE**	**−** **CSE**	**+CSE**
**Economic Input**				
Price/kg feed	2	2	2	2
Chicks price	4.5	4.5	4.5	4.5
Feeding cost/bird	6.136	5.904	5.574	6.398
Labor cost/bird	0.175	0.175	0.175	0.175
Water cost/bird	5.96	5.96	5.96	5.96
Transportation cost/bird	0.21	0.21	0.21	0.21
Vaccine and medication cost/bird	1.13	1.13	1.13	1.13
Total cost/bird	18.111	17.879	17.549	18.373
**Economic Output**				
Chicken price/bird	22.638	23.232	15.752	19.228
Manure revenue/bird	0.55	0.55	0.55	0.55
Total revenue/bird	23.188	23.782	16.302	19.778
Profit/bird	5.077	5.903	−1.247	1.405
Economic efficiency	0.28	0.33	−0.07	0.08
Relative economic efficiency	100	117.8	−25.3	27.3

All the cost and prices listed are in Saudi Arabian riyal (1 USD = 3.76 SAR) according to the present market prices.

## Data Availability

The data presented in this study are available on request from the corresponding author.
